# Well-being and health-related quality of life in new-generation migrant workers in Zhejiang province, China

**DOI:** 10.1186/s12955-019-1193-y

**Published:** 2019-07-12

**Authors:** Haiyan XING, Wei YU, Weiqian CHEN, Xiaoqi CHENG

**Affiliations:** 1 Department of Nursing, School of Medicine, Shaoxing University, No.900 Chengnan Avenue Shaoxing, Zhejiang Province, 312000 China; 2 Institute of Epidemiology, Shaoxing Keqiao District Center for Disease Control and Prevention, No.1356 Xingyue Road, Shaoxing Keqiao, Zhejiang Province, 312030 China

**Keywords:** Well-being, Health-related quality of life, new-generation migrant workers

## Abstract

**Background:**

The purpose of this study is to evaluate the reliability and validity of the multiple happiness questionnaire (MHQ) in new-generation migrant workers (NGMW), to compare the difference of well-being and Health-Related Quality of Life (HRQOL) in NGMW with first-generation migrant workers (FGMW) and urban workers (UW), and to explore the relationship between well-being and HRQOL and analyze influential factors to well-being in NGMW in Zhejiang province, China.

**Methods:**

By stratified sampling, 542 NGMW, 226 FGMW and 200 UW had completed the questionnaires in 2018. Cronbach’s alpha coefficient (a) for internal consistency of the multiple happiness questionnaire (MHQ) was used. Factor analysis was applied for construct validity. Scores of well-being and HRQOL were compared between NGMW and control groups. Spearman’s correlation was performed to clarify the relationship between well-being and HRQOL in NGMW. Multiple linear regression analytical methods were used to adjust confounding effects and to identify the variables that were associated with well-being.

**Results:**

MHQ had good internal consistency (Cronbach’s alpha overall was 0.960, subscales ranged from 0.754 to 0.957) and structural validity based on factor analysis. Except for life satisfaction and altruism commitment, there was a positive correlation between well-being and HRQOL in NGMW. There were significant differences in psychological well-being (PWB), health concern, subjective vitality, physical component summary (PCS) and mental component summary (MCS) between NGMW and FGMW. Compared to UW, NGMW’s general well-being (GWB), subjective well-being (SWB), life satisfaction, positive relation and altruism commitment scores were lower and their negative affect was higher. The GWB score was related to MCS, PCS, self-reported social status, marital status, age and monthly income.

**Conclusion:**

The results suggest that the MHQ is a reliable and valid measure for well-being in NGMW. There is a significant difference in well-being and HRQOL between NGMW and control groups. Well-being is higher in NGMW than in FGMW, but is lower than in UW. Well-being is related with HRQOL and may be affected by MCS, PCS, self-reported social status, marital status, age and monthly income in NGMW.

## Introduction

According to the 2016 Report on China’s Migrant Population Development [1], the number of migrant population reached 253 million in 2015 in China, more than half of which were the new-generation migrant workers (NGMW).The migrant workers had made tremendous contributions to the rapid development of the local economy in urban areas. Although many industries in the city had become increasingly dependent on the migrant workers, due to the restrictions of the household registration system, they could not enjoy the same treatment as the urban residents. On the other hand, low educational degree and income of migrant workers aggravated the dilemma and they were on the edge of the social network [2].

New-generation migrant workers were born in the 1980s and began to flow around the 1990s. Compared with the first-generation migrant workers, the group characteristics mainly included that they had higher education levels, had no or less family burden, went out directly after graduating from school and had few farming experiences, they paid more attention to changing lifestyles and pursued better development opportunities [3].Some researchers pointed out that the main dilemmas faced by the NGMW include personal marriage, identity, career development and the barriers arising from economic, quality and social welfare [2].

The pursuit of happiness is a long-enshrined tradition that has recently become the cornerstone of the American Positive Psychology movement [4]. With the acceleration of China’s reform and opening up process, urbanization and the living standards of residents have continuously improved. The improvement of life satisfaction and quality of life and happiness will become the goal pursued by the residents. “Pursuit of happiness” and realizing the goal of life are the main purpose of the NGMW. Studies have shown that the subjective well-being of migrant workers is not only lower than that of urban residents, but also lower than that of rural residents, mainly because they have a strong sense of relative deprivation [5].

The contemporary studies of happiness in the developed world demonstrate the change in the focus of research interest from objective well-being to subjective well-being and from the economic rationale of happiness to the psychological rationale of happiness [6]. Numerous studies have found that although subjective well-being (SWB) and psychological well-being (PWB) show many differences, they have shown a trend of mutual integration. For a better and more comprehensive understanding of happiness, integrating SWB and PWB will be a major trend in the study of future happiness [7]. Miao YJ developed a comprehensive scale (Multiple Happiness Questionnaire, MHQ) in Chinese population, which integrated SWB and PWB [8]. MHQ was a reliable and valid measure for well-being [8–10]. Most of these investigations suggested that the main influential factors to well-being in migrant workers included income level, age, education, social support, Health-Related Quality of Life (HRQOL) and so on [5, 11, 12]. Previous studies reported that there were significant positive correlation between HRQOL and well-being [13, 14].

In this study, well-being of NGMW was assessed by comparing with first-generation migrant workers (FGMW) and urban workers (UW). The relationship of well-being and HRQOL and main influential factors in NGMW on well-being were analyzed by investigation.

## Methods

### Subjects and procedure

For study purpose, migrant workers are defined as people who migrate from rural to urban areas. Zhejiang province is well-known throughout the country as the Eastern coastal city in China. Its gross domestic product(GDP)ranks fourth in recent years around the country and attracts a large number of migrant workers, the number reaches above 19 million.

Data was obtained from cross-sectional survey in 2018 in Zhejiang province. The target population comprised migrant workers and urban dwellers from three cities aged above 15, and were randomly selected by stratified sampling technique. At first, three stratifications were divided by economic level in Zhejiang province (3, 4, 4 cities respectively). One city was chosen from each stratification: Hangzhou, Shaoxing and Quzhou. In each city a list of work units where migrant workers gathered was drawn up. Some typical work units were selected such as manufacturing industry in Hangzhou, textile industry in Shaoxing, construction industry in Quzhou.

The study included participants who were above 15 years old and consisted of three groups. The first and second groups comprised migrant workers, defined as individuals who held rural household registration (hukou) and worked at an urban destination for over 3 months. According to age, there consisted two groups: the first generation and new generation migrant workers (birth year before or after 1980). The third group in this study comprised urban workers (birth year after 1980) who were born in city and held urban household registration. Migrant workers and urban workers were drawn from the same work units in three cities. In each work unit, all of the workers present on the same day were recruited into the study. The valid questionnaires of the three groups were 542, 226 and 200 respectively and the average response rate reached 94.9%.

### Measure of well-being and quality of life

Well-being was assessed by the Multiple Happiness questionnaire (MHQ). The MHQ is a 50-item scale with 9 domains: life satisfaction (5 items), positive affect (6 items), negative affect (6 items), health concern (5 items), subjective vitality (6 items), self-worth (5 items), person growth (9 items), positive relation (3 items) and altruism commitment (5 items) [8]. The Subjective Well-Being (SWB) comprised of life satisfaction, positive affect and negative affect. The Psychological Well-Being (PWB) comprised of the health concern, subjective vitality, self-worth, person growth, positive relation and altruism commitment. Within the scale, all items are in a 7-point response scale. Item 12 and item 14 are reverse-scored. The General Well-Being (GWB) score is the total score from the nine domains. The coefficient of internal consistency is among 0.67~0.91, split-half reliability is among 0.62~0.88, the scale is appropriate for the Chinese population. The higher score indicates better happiness [8].

Health-related quality of life was measured by the 12-item Short Form Health Survey (SF-12). The SF-12 is extracted from the SF-36 and is a shorter alternative of the SF-36 instrument that includes 12 questions and 8 domains: physical functioning (2 items), role physical (2 items), bodily pain (1 item), general health (1 item), vitality (1 item), social functioning (1 item), role emotional (2 items) and mental health (2 items). Each dimension is scored on a 0–100 scale with 0 and 100 corresponding to worst and best HRQOL respectively. The eight domains can be summarized in two summary scores of physical and mental component summary (PCS and MCS) [15, 16]. The scale was translated to Chinese and validated previously, the reliability and validity were excellent in floating population (Cronbach’s a = 0.84, convergent validity experiment rate and discriminate validity experiment rate were 100%) [17].

### Statistical analysis

Statistical analyses were performed using SPSS version 18.0 and Mplus version 6.11 software, including Chi-square test for gender and marital status, Mann-Whitney U for other sociodemographic characteristics and independent samples t-test for age, Well-Bing and HRQOL scores. Reliability and the internal consistency for MHQ were measured using Cronbach’s alpha coefficient and an alpha equal to or greater than 0.70 was considered satisfactory [18]. Exploratory factor analysis (EFA) and confirmatory factor analysis (CFA) were used to measure the factor structure of MHQ. Spearman’s correlation was performed to identify the relationship between well-being and HRQOL. Multiple linear regression was performed to adjust confounding effects and to assess the impact of related variables. Variables in the model included age, gender, education, marital status, monthly income, self-reported social status, PCS and MCS. Monthly income was classified into three levels including low (< 3100 yuan), medium (3100~5000 yuan) and high (≥5000 yuan) based on percentiles. Education, marital status and self-reported social status were also classified into three levels (Table 1), so two dummy variables were established respectively. Gender and education were excluded by stepwise regression (*p* > 0.05). The variance inflation factor (VIF) of all variables was less than 2.0 in final model based on collinearity diagnositics.

**Table 1:** Sociodemographic characteristics of new-generation migrant workers and control group

		NGMW n (%)	FGMW n (%)	UW n (%)	*P*_*1*_	*P*_*2*_
*n*		542	226	200		
Age (mean ± standard deviation)		28.6 ± 0.2	46.6 ± 0.4	29.1 ± 0.4	< 0.001^a^	0.337 ^a^
Gender	Male	304 (56.1)	153 (67.7)	109 (54.5)	0.003^b^	0.699^b^
Education	Primary	45 (8.3)	56 (24.8)	4 (2.0)	< 0.001^c^	
	Secondary	208(38.4)	98 (43.4)	26 (13.0)		< 0.001^c^
	Higher	289 (53.3)	72 (31.9)	170 (85.0)		
Marital status	Single	166 (30.6)	8 (3.5)	61 (30.5)	< 0.001^b^	
	Married/cohabiting	353 (65.1)	187 (82.7)	137 (68.5)		0.090^b^
	Divorced/separated/widowed	23 (4.2)	31 (13.7)	2 (1.0)		
Monthly income	Low	114(21.0)	58(25.7)	61(30.5)		
	Medium	234(43.2)	83(36.7)	70(35.0)	0.719^c^	0.112^c^
	High	194(35.8)	85(37.6)	69(34.5)		
Self-reported social status	Low	110 (20.3)	50 (22.1)	38 (19.0)		
	Medium	381 (70.3)	150 (66.4)	137 (68.5)	0.997^c^	0.346^c^
	High	51 (9.4)	26 (11.5)	25 (12.5)		

*P*_*1*_: NGMW vs. FGMW *P*_*2*_*:* NGMW vs. UW

^a^: independent samples t-test ^b^: Chi-square test ^c^: Mann-Whitney U

## Results

### Sociodemographic characteristics

Data was obtained from 542 new-generation migrant workers (NGMW), 226 first-generation migrant workers (FGMW) and 200 urban workers (UW). Their sociodemographic characteristics are shown in Table 1. Significant differences in age, gender, education and marital status were found between NGMW and FGMW. The FGMW were older, with a larger proportion of men, less well educated and less likely to be single (*p* < 0.05). Compared to UW, NGMW were less well educated (*p* < 0.05). Migrant workers originated from 26 of the 31 Chinese provinces: 167 (21.7%) were from Zhejiang, and 417 (54.3%) were from poorer inland provinces, including Guizhou, Anhui, Jiangxi, Sichuan, Henan and Hunan.

### MHQ reliability and validity assessment

Internal consistency of MHQ in NGMW was measured using the Cronbach’s alpha coefficient (a). Cronbach’s alpha for the subscales ranged from 0.754 to 0.957, and the alpha for the total score was 0.960 (Table 2).

**Table 2:** Internal consistency and correlation coefficients of MHQ and SF-12 in NGMW

	GWB	SWB	PWB	LS	PA	NA	HC	SV	SW	PG	PR	AC
Cronbach’s a	0.960	0.870	0.957	0.906	0.833	0.879	0.924	0.916	0.885	0.754	0.894	0.913
Correlation coefficients (r)												
PCS	0.207^*^	0.227^*^	0.167^*^	0.070	0.233^*^	−0.146^*^	0.186^*^	0.117^*^	0.132^*^	0.206^*^	0.093^*^	0.033
MCS	0.252^*^	0.270^*^	0.217^*^	0.134^*^	0.164^*^	−0.266^*^	0.177^*^	0.122^*^	0.209^*^	0.266^*^	0.120^*^	0.082

General Well-Being(GWB), Subjective Well-Being (SWB), Psychological Well-Being (PWB), Life satisfaction (LS), Positive affect (PA), Negative affect (NA), Health concern (HC), Subjective vitality (SV), Self-worth (SW), Person growth (PG), Positive relation (PR), Altruism commitment (AC),^*^p < 0.05

Kaiser-Meyer-Olkin measure of MHQ showed that the data met the conditions of factor analysis, which was above 0.7 and *p* < 0.05 (KMO = 0.939, *P* < 0.001). Nine factors were extracted based on the principle of Initial Eigenvalues > 1. The nine-factor structure jointly accounted for 71.59% of the variance based on exploratory factor analysis.

The nine-factor model was specified and tested by confirmatory factor analysis. The results provided a good fit to the data lending support to the original hypothesized structure of the questionnaire with comparative fit index (CFI) =0.909, Tucker-Lewis index (TLI) =0.902, standardized root mean square residual (SRMR) =0.049, root mean square error of approximation (RMSEA) =0.051, 90%CI RMSEA = 0.048 to 0.053.

### Well-being and HRQOL between NGMW and control groups

There were significant differences in GWB, SWB, PWB, NA, HC, SV, PR and AC between NGMW and control groups before the confounding effects were adjusted (*P* < 0.05, Table 3). After confounding effects were adjusted, PWB, HC and SV scores of NGMW were higher than that of FGMW, however compared to urban workers their GWB, SWB, LS, PR and AC scores were lower and NA was higher (*P* < 0.05, Table 3).

**Table 3:** Distribution of Well-Being and HRQOL scores in NGMW and control groups (mean ± standard deviation)

	NGMW	FGMW	UW	*P*_1_^a^	*P*_2_^a^	*P_1_^b^	^*^ *P*_2_^b^
Well-Being							
GWB	4.85 ± 0.76	4.72 ± 0.71	4.97 ± 0.84	0.024	0.064	0.065	0.027
SWB	4.83 ± 0.84	4.80 ± 0.80	4.99 ± 0.94	0.593	0.038	0.594	0.018
PWB	4.85 ± 0.84	4.67 ± 0.80	4.96 ± 0.92	0.006	0.138	0.026	0.057
LS	4.32 ± 1.26	4.40 ± 1.11	4.48 ± 1.28	0.378	0.132	0.895	0.022
PA	4.67 ± 1.21	4.55 ± 1.21	4.68 ± 1.27	0.212	0.948	0.452	0.954
NA	2.55 ± 1.30	2.61 ± 1.23	2.20 ± 1.15	0.574	0.001	0.907	0.010
HC	5.29 ± 1.11	5.00 ± 1.13	5.28 ± 0.08	0.001	0.924	0.024	0.856
SV	4.72 ± 1.13	4.32 ± 1.19	4.87 ± 1.07	< 0.001	0.106	0.002	0.066
SW	5.05 ± 1.04	5.00 ± 1.04	5.15 ± 1.10	0.518	0.270	0.288	0.182
PG	4.58 ± 0.81	4.53 ± 0.81	4.62 ± 0.90	0.458	0.535	0.332	0.201
PR	4.97 ± 1.22	4.81 ± 1.15	5.25 ± 1.13	0.080	0.005	0.174	0.003
AC	4.79 ± 1.15	4.63 ± 1.05	4.99 ± 1.16	0.072	0.033	0.177	0.015
HRQOL							
PCS	50.40 ± 6.66	46.43 ± 7.57	50.99 ± 6.58	< 0.001	0.291	< 0.001	0.843
MCS	46.51 ± 7.94	48.26 ± 7.11	47.13 ± 7.87	0.005	0.361	0.006	0.168

Physical component summary (PCS), Mental component summary (MCS)

*P*_*1*_: NGMW vs. FGMW *P*_*2*_*:* NGMW vs. UW

^*^P_1_: adjust in Gender, Education, Marital status, Monthly income;

^*^P_2_: adjust in Education, Monthly income

^a^: independent samples t-test ^b^: multiple linear regression

Significant differences in PCS and MCS were found between NGMW and FGMW (*P* < 0.05, Table 3). PCS score of new-generation migrant workers was higher than that of FGMW, however MCS score was lower (*P* < 0.05, Table 3).

### Well-being and influential factors in new-generation migrant workers

Except for LS, AC and PCS, AC and MCS, the correlation coefficients were significant between well-being and HRQOL in NGMW (*P* < 0.05, Table2).

The GWB score was related to MCS, PCS, self-reported social status, marital status, age and monthly income. Well-Being was positively influenced by MCS and PCS. Compared to low social status, medium and high social status had better well-being. Married and cohabiting individuals scored higher on GWB than single, divorced, separated and widowed individuals. GWB was negatively related with age. High, income had positive influence on well-being compared to low income (Table 4).

**Table 4:** Variables associated with Well-Being (GWB) in NGMW, revealed by multiple linear stepwise regression

	*B*	*Beta*	*t*	*p*	95%*CI* for *B*	*VIF*
Constant	2.941		8.2148	< 0.001	2.237~3.644	
MCS	0.025	0.261	6.439	< 0.001	0.017~0.032	1.012
PCS	0.024	0.208	5.053	< 0.001	0.014~0.033	1.040
Self-reported social status (control = low)						
Medium	0.224	0.135	2.906	0.004	0.072~0.375	1.332
High	0.414	0.157	3.317	0.001	0.169~0.659	1.379
Marital status (control = married/cohabiting)						
Single	−0.325	−0.198	−4.178	< 0.001	−0.478~ − 0.172	1.380
Divorced/separated/ widowed	− 0.419	− 0.117	−2.851	0.005	− 0.708~ − 0.130	1.028
Age	−0.022	− 0.164	−3.490	0.001	− 0.035~ − 0.010	1.349
Monthly income (control = low)						
Medium	0.072	0.048	0.868	0.386	−0.092~0.237	1.844
High	0.253	0.162	2.908	0.004	0.082~0.424	1.908

*B*: unstandardized coefficients, *Beta*: standardized coefficients

*VIF*: variance inflation factor, Multiple Correlation Coefficient, *R* = 0.453

## Discussion

Previous studies showed that the Multiple Happiness questionnaire (MHQ) has good reliability and validity among different population such as college students, middle school students and elderly population [8–10]. Our study showed that the reliability of the overall scale, two summary scores and nine domains were very good (Cronbach’s a coefficient was above 0.7). With regard to construct validity, the subscores of MHQ were found to be satisfactory. Nine factors extracted by EFA were consistent with the theoretical structure and there was a good fit for the data of the questionnaire based on CFA (CFI = 0.909, TLI = 0.902, SRMR = 0.049, RMSEA = 0.051). For the CFI and TLI, the recommended cut-off for acceptable values are > 0.90. The model is well fitting for SRMR< 0.08. For the RMSEA, values of < 0.05 indicate a close fit and values below 0.11 are an acceptable fit [19, 20]. So MHQ is a reliable and valid measure for well-being in new-generation migrant workers.

Our results indicated that NGMW’s scores of PWB, HC and SV were much higher than FGMW’s. Compared to urban workers, NGMW’s GWB, SWB, LS, PR and AC scores were lower and NA score was higher (Table 3). NGMW had many differences with FGMW in their family environment, age, education, marital status, personal traits and farming experience. In addition, compared to FGMW, NGMW show new features in their work and life, such as they hope to improve their quality of life, realize their ideals, learn more knowledge and technology rather than survive and improve the family’s economic situation. NGMW also desire to get decent work and be close to the citizens rather than only earn more money than farming. Most NGMW wanted to live in city long time and gain long-term development rather than return to original countryside sooner or later [21]. Well-being of NGMW was higher than that of FGMW, especially in psychological well-being (PWB), health concern (HC) and subjective vitality (SV). NGMW had a strong sense of identity with the city, and their lifestyles were close to those of urban residents, greatly reducing the incompatibility of FGMW to urban life. However, NGMW’s general well-being (GWB), subjective well-being (SWB), life satisfaction (LS), positive relation (PR) and altruism commitment (AC) scores were lower and their negative affect (NA) was higher than that of urban workers. Although NGMW have received better education than FGMW, their social life circle is still narrow and it is more difficult to get social support than urban workers, which affect the happiness of NGMW. Due to the gap of high consumption of urban life and NGMW’s low income, they are vulnerable to feel “relative deprivation” [22]. Some of them suffered prejudice and discrimination from urban dwellers and produced stronger negative emotions.

There were significant differences in HRQOL (PCS and MCS) between NGMW and FGMW whether or not the confounding factors were adjusted (Table 3). Compared to FGMW, NGMW’s PCS score was higher, however MCS score was lower. Li et al. found that with the increase of age in city dwellers, both male and female, the PCS scores showed a downward trend, but their MCS scores showed an upward trend, which might be due to the greater social, work and life pressure on young and middle-aged people [16]. Except for LS and AC, correlation was found between well-being and HRQOL (Table 2), which was similar with other population study [13, 14, 23]. There was a negative correlation between NA and HRQOL, while other domains of MHQ (except for LS and AC) had positive correlation with PCS and MCS. The present results revealed the main factor that influenced NGMW’s well-being was HRQOL. The effect of MCS and PCS ranked first and second in the included variables (Table 4). Well-being is a positive subjective and psychological experience of self-existence and development, which is caused by the combination of objective conditions and demand value of individuals. Health-related quality of life directly determines the overall satisfaction of individuals in their own lives. Well-being and HRQOL are consistent from a certain point of view, they both assess health status and satisfaction based on subjective feelings. Similar studies showed that mental health and physical function were influencing factors of subjective well-being of the elderly [24]. There was very little research on the relationship between the well-being and HRQOL in NGMW. Compared to low social status, medium and high social status had positive influence on well-being. Xu et al. revealed that social status not only affected happiness, but also contributed more to the happiness of Chinese residents than income. The impact of social status on well-being increased as income increased [25]. GWB scores of married and cohabiting individuals were higher than those of single, divorced, separated and widowed individuals, which was similar to previous studies [26]. Stable marriage relationships often provided more material and emotional support. A negative relationship was found between age and well-being, and this result may be different from other studies [26, 27]. The discrepancy may be attributable to the different age ranges. NGMW were born after 1980 year, with different age ranges for different investigation years. NGMW were 15–38 years old in this study rather than the usual 15–30 or other ranges. Well-being was higher in high income workers compared to low income population. Although as people’s income increased, the impact of income on happiness gradually declined, economic factors still had played a fundamental role in the well-being of residents and higher income groups could experience more happiness [25, 26]. Inequality in income will lead to a sense of inferiority in resources and bring a strong sense of deprivation, which have a negative impact on happiness [26]. Nearly two-thirds population in high-income NGMW believed that raising income levels was one of the ways to improve happiness [28], the primary reason why NGMW left from rural to city was to increase the income to improve their quality of life. However their low level of skills made them earn lower salaries than the city dwellers. Some migrant workers sometimes suffered unfair event from employer such as “arrears of wages” and “deduction of wages” [26]. So the government should strengthen supervision mechanism to ensure that migrant workers receive legal income on schedule.

In order to further to improve the happiness and quality of life of the NGMW, government should improve the social security system and basic medical facilities to meet the needs of health and sanitation. The income of migrant workers should gradually increase, narrow the gap between urban workers and achieve equal pay for equal work. Better implementation of relevant laws should be strengthened. The society and the community work together to create a social atmosphere of equality, harmony and mutual assistance, and promote social integration.

### Limitations

The study only sampled three cities in this province, although it had a large sample size, the results were not applicable to all aspects and could not be generalized to whole migrant workers in China. It was difficult to establish cause and effect relationship between well-being and influential factors based on a cross sectional study. Other factors such as social support and sudden positive or negative events that were known as influencing factors on well-being, were not measured in this research.

## Conclusions

The study demonstrated an acceptable reliability and validity of MHQ in specific population. The findings of this study highlighted the differences of well-being and HRQOL between the new-generation migrant workers and control groups (first-generation migrant workers and urban workers). This analysis provided additional evidence supporting that well-being in NGMW was higher than in FGMW, but was lower than in UW. A positive correlation was found between well-being and HRQOL. The main influential factors on well-being were MCS, PCS, self-reported social status, marital status, age and monthly income.

## Data Availability

Please contact author for data requests.

## Abbreviations

CFA: Confirmatory factor analysis
EFA: Exploratory factor analysis
FGMW: First-generation migrant workers
GWB: General well-being
HRQOL: Health-related quality of life
MCS: Mental component summary
MHQ: Multiple happiness questionnaire
NGMW: New-generation migrant workers
PCS: Physical component summary
PWB: Psychological well-being
SWB: Subjective well-being
UW: Urban workers
VIF: variance inflation factor
